# Physics‐Based Spatial Oversampling of TROPOMI NO_2_ Observations to US Neighborhoods Reveals the Disparities of Air Pollution

**DOI:** 10.1029/2025GH001423

**Published:** 2025-08-16

**Authors:** Xiaomeng Jin, Zaina Merchant, Kang Sun

**Affiliations:** ^1^ Department of Environmental Sciences Rutgers, The State University of New Jersey New Brunswick NJ USA; ^2^ Department of Civil, Structural and Environmental Engineering University at Buffalo Buffalo NY USA

**Keywords:** remote sensing, NO_2_, environmental justice, TROPOMI, air pollution disparity, spatial oversampling

## Abstract

Satellite observations provide continuous and global coverage observations of air pollutants, widely used to inform health impacts and air pollution disparities. Linking satellite retrievals with socioeconomic or health data involves matching the irregularly shaped satellite observations with administrative units. Here, we develop a physics‐based approach to spatially oversample nitrogen dioxide (NO_2_) retrievals from TROPOspheric Monitoring Instrument (TROPOMI) directly to United States (US) neighborhoods (i.e., block groups). The physics‐based oversampling approach considers each satellite pixel as a sensitivity distribution, meaning that satellite instruments are more sensitive to the neighborhoods at the center than at the edge of the observations. We show that directly oversampling satellite observations to administrative shapes is a more accurate and computationally efficient approach than the commonly used gridding approaches, and it is advantageous for shorter temporal windows. Combining the newly developed NO_2_ data set with demographic data, we find widespread racial/ethnic and income‐related NO_2_ disparities across the US. NO_2_ disparities are even more pronounced during the most polluted days, suggesting greater acute health effects for overburdened communities. We expect that the resolution‐adaptive, neighborhood‐level, and GIS‐compatible NO_2_ data set would lower barriers of the public to access and interpret satellite observations, facilitating the actionable applications of satellite observations.

## Key Points

- We develop a physics‐based approach to spatially oversample TROPOspheric Monitoring Instrument NO_2_ retrievals directly to United States neighborhoods
- We find widespread racial/ethnic and income‐related NO_2_ disparities across urban areas of contiguous US
- NO_2_ disparities increase during the most polluted days

## 1 Introduction

Nitrogen dioxide (NO_2_) is a gaseous air pollutant linked to respiratory, metabolic and cardiovascular issues (Atkinson et al., 2013; T.‐M. Chen et al., 2007; Meng et al., 2021; Zang et al., 2021), and is a precursor of ground‐level ozone and fine particulate matter that are leading contributors to air pollution related premature deaths (Murray et al., 2020). Over the U.S., NO_2_ is regulated as one of the six criteria air pollutants under the National Ambient Air Quality Standards. NO_2_ is a primary air pollutant, formed mainly from fossil fuel combustion in electric generating units, high‐temperature processes at industrial sources and operation of motor vehicles (U.S. EPA, 2023), and a small fraction nationally and annually is emitted from natural processes such as lightning (Murray, 2016), soil (Hudman et al., 2010), and biomass burning (Jin et al., 2021, 2023). High levels of NO_2_ are found near roadways and industrial facilities, which are often located near marginalized communities (Mohai et al., 2011; Rowangould, 2013). Recent studies have highlighted significant disparities in NO_2_ exposure across the United States (US), disproportionately affecting racial and ethnic minority communities (Demetillo et al., 2021; Hrycyna et al., 2022; Kerr et al., 2021; Y. Wang et al., 2023). As a result of emission controls under the Clean Air Act, NO_2_ levels have significantly declined across the US (Duncan et al., 2016), but the NO_2_ disparities persist and even get worse over time (Y. Wang et al., 2023).

Mapping disparities of air pollution has been challenging due to the sparse ground‐based air quality monitors, which are also disproportionally distributed (Kelp et al., 2023). Remote sensing observations provide global, full‐coverage, long‐term observations of atmospheric composition for decades, which show promise in quantifying the environmental disparities (Colmer et al., 2020; Demetillo et al., 2021; Hrycyna et al., 2022; Kerr et al., 2021; Sayyed et al., 2024). In October 2017, the TROPOspheric Monitoring Instrument (TROPOMI) was launched into space, offering an unprecedented view to detect urban NO_2_ pollution at a fine spatial resolution of 3.5 × 5.5 km^2^ (Veefkind et al., 2012). TROPOMI NO_2_ retrievals provide observational evidence of the persistent NO_2_ disparities over major US cities (Demetillo et al., 2021; Dressel et al., 2022; Kerr et al., 2021).

The pixel geometry of TROPOMI observations varies with viewing angles. Generally, Level‐2 satellite observations from multiple orbits are projected and averaged to a regularly spaced Cartesian latitude‐longitude grid, known as a “Level‐3” product. By aggregating longer‐term observations into a common grid, Level‐3 products not only reduce the observational noise of individual observations, but also help achieve higher spatial resolution by sampling a given target repeatedly with varying geometries, an approach referred to as spatial oversampling. Various spatial oversampling algorithms have been developed. A commonly used approach considers satellite observations as polygons with four corners. The value for the target grid cell is determined as the mean of the observations weighted by the overlapping area between target grid and satellite pixel polygon, referred to as area‐weighted spatial oversampling (AWO), which has been adopted by many operational Level‐3 products (Duncan et al., 2016; Foy et al., 2015; Goldberg et al., 2017; Jin et al., 2020). This approach assumes that satellite instrument is uniformly sensitive to the scene inside the pixel polygon and has no sensitivity outside the polygon. For a push‐broom imaging spectrometer like TROPOMI, the field of view (FoV) is not quadrangular, but rather continuous as a Gaussian‐shape sensitivity distribution that overlaps with the FoV of neighborhood pixels (Graaf et al., 2016). Sun et al. (2018) developed a physics‐based oversampling algorithm which considers satellite observations as a sensitivity distribution, instead of assuming satellite pixels as polygons. They show that the physics‐based oversampling approach is advantageous for smaller temporal windows and better captures the local spatial gradient of NO_2_ (Demetillo et al., 2021; Sun et al., 2018).

The target of the above mentioned spatial oversampling approaches is a regularly spacing grid cell, which has shown to be useful for visualizing the spatial and temporal variations of air pollution, and comparison with model simulations. However, socioeconomic and health surveys are conducted at administrative levels, which differ from the gridded data. Linking satellite‐retrieved air pollution data with socioeconomic or health data involves matching the irregularly shaped satellite observations with administrative units (e.g., block group, census tract). Previous applications of satellite‐based NO_2_ to environmental justice studies involve an additional step of assigning the gridded NO_2_ to administrative units by searching for the nearest grid (Kerr et al., 2021; Y. Wang et al., 2023), which could cause mismatch issues if the administrative units have irregular shapes or when their areas are smaller than the grid size (Kerr et al., 2021). Over the US, 43% block groups are smaller than 1 km^2^ (Figure S1 in Supporting Information S1), which is finer than the grid size of most gridded NO_2_ products (0.01° or coarser). Also, large numbers of observations are needed to achieve the resolution of 0.01°, so generally only monthly or annual‐level products are publicly available (Goldberg, 2024), limiting the applications of TROPOMI NO_2_ to study the short‐term variability of air pollution. Here, instead of using gridded Level‐3 data, we apply the physics‐based spatial oversampling approach directly to administrative units at the block group level. The resulting block‐group TROPOMI NO_2_ data set is available in tabular or GIS shapefile formats, directly compatible with the formats of most socioeconomic, demographic and health data. Using the newly developed TROPOMI NO_2_ data set, we evaluate the racial/ethnic and income‐related disparity of NO_2_ pollution over the contiguous US (CONUS), and the changes of NO_2_ disparity during polluted days when NO_2_ tends to be most harmful to human health. We demonstrate the neighborhood‐level, GIS‐compatible NO_2_ data set can lower the barriers for the communities to access and interpret satellite data, which will facilitate interdisciplinary applications of satellite data in areas such as public health, socioeconomics, decision‐making, and environmental justice.

## 2 Materials and Methods

### 2.1 Spatial Oversampling of TROPOMI NO_2_ Observations

TROPOMI is a push‐broom hyperspectral spectrometer launched by the European Space Agency on 13 October 2017 for the Copernicus Sentinel‐5 Precursor satellite mission. TROPOMI provides afternoon (∼1:30 p.m. local time) observations with a spatial resolution of 5.5 × 3.5 km^2^ at nadir in the ultraviolet and visible spectra. We obtain 5‐year (2019–2023) daily Level‐2 TROPOMI retrievals of NO_2_ tropospheric column (version 2.4) from the Tropospheric Emission Monitoring Internet Service (TEMIS) hosted by the Royal Netherlands Meteorological Institute (Copernicus Sentinel‐5P, 2021; Geffen et al., 2022). We use the reprocessed (RPRO) product before July 2022, and the offline (OFFL) product after that.

We include TROPOMI NO_2_ observations with quality assurance values greater than 0.75 to exclude problematic retrievals. The screened Level‐2 NO_2_ data are aggregated to U.S. block groups using two spatial oversampling approaches: area‐weighted (hereafter AWO‐BG) and physics‐based Gaussian (hereafter PGO‐BG) spatial oversampling approaches. For the AWO‐BG approach, each satellite pixel is considered as a polygon with four corners (available from TROPOMI products). For each block group, we search for all screened satellite pixels that overlap with the target block group, and calculate the overlap area (*wa*_*i,x*_) between the target block group *x* and each satellite pixel polygon *i*, and the resulting average NO_2_ column (${\bar{\Omega }}_{x,t}$) for each block group for a given temporal window *t* is calculated as Equation 1:

(1) $${\bar{\Omega }}_{x,t}=\frac{\sum_{t}\sum_{I}{wa}_{i,x}\times \Omega_{i}}{\;\sum_{t}\sum_{I}{wa}_{i}}$$

where Ω_i_ is the retrieved NO_2_ tropospheric column density of each observation that overlaps with the block group *x*. Both the target block group and the satellite pixel are defined as geometric objects (i.e., polygons), and the intersection areas can be efficiently calculated through vector‐based spatial analysis. The intersection areas are calculated using Shapely (Gillies, 2007), a widely used Python library for manipulation and analysis of geometric objects.

The PGO‐BG approach considers satellite observations as a sensitivity distribution rather than a polygon, meaning that satellite instruments are more sensitive to the neighborhoods at the center than at the edge of the observations. For each observation, we define a 2‐D super Gaussian spatial response function (Equation 2) to describe the sensitivity of satellite instrument (Graaf et al., 2016; Sun et al., 2018):

(2) $$S\left(x,y\right)=\exp\left(-\left({\left|\frac{x}{w_{x}}\right|}^{n}+{\left|\frac{y}{w_{y}}\right|}^{m}\right)\right)$$

where

(3) $$w_{x}=\frac{{\text{FWHM}}_{x}}{{2\left(\log2\right)}^{1/n}}$$

(4) $$w_{y}=\frac{{\text{FWHM}}_{y}}{{2\left(\log2\right)}^{1/m}}$$

where *x* and *y* are distances to the center of the ground FoV in orthogonal directions, transformed by geometric projections of the along‐ (*x*) and across track (*y*) directions. FWHM_*x*_ and FWHM_*y*_ are the full widths at half maximum of the spatial response function *S*(*x*,*y*) in the along‐ and across‐track directions. The exponential terms *n* and *m* determine the shape of the Gaussian distribution function, and we use *n =* 2 and *m* = 4 for TROPOMI (Graaf et al., 2016; Sun et al., 2018). Each satellite observation corresponds to a distinct spatial response function. For each block group of interest, we calculate the sum of *S*(*x*,*y*) that overlaps with the block group (hereafter *wg*). As the block groups are defined as polygons, we rasterize each block group into a 10 × 10 grid to calculate the corresponding spatial response function, where the extent of the grid is defined by the minimum and maximum latitude and longitude of the block group. The long‐term average NO_2_ for a given temporal window *t* is calculated as Equation 5:

(5) $${\bar{\Omega }}_{x,t}=\frac{\sum_{t}\sum_{I}{wg}_{i,x}\times \Omega_{i}}{\;\sum_{t}\sum_{I}{wg}_{i}}$$

**Figure 1:**
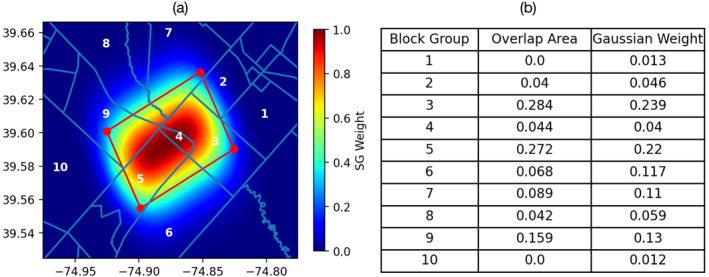
(a) Illustration of the 2‐D Gaussian spatial response function of a single satellite observation overlaid with the administrative boundaries of 10 block groups in the satellite field of view. The response function is projected to the Cartesian space and normalized by the sum. (b) Comparison of the weight assigned to each block group (labeled in a) based on overlap areas (AWO‐BG) or the Gaussian weight (PGO‐BG). The area is normalized by the area of the satellite pixel polygon.

Figure 1 shows an example of the spatial response curve projected to the Cartesian space overlaid with the administrative boundaries of 10 block groups in the satellite FoV. The table on the right compares the weight difference between AWO‐BG and PGO‐BG. As satellite instrument is more sensitive to the radiation signals at the center of the FoV than the edge of the FoV, block groups at the center of the FoV (e.g., Block Groups 4,5) carry more weight. Both AWO‐BG and PGO‐BG approaches give higher weight for block groups with larger overlap areas with the observations. Since AWO‐BG assumes that the satellite signal is fully contained within each observation polygon, block groups located outside the polygon boundaries receive no weight under the AWO‐BG approach (e.g., Block Groups 1 and 10), but the PGO‐BG method assigns small but non‐zero weights to these block groups.

For comparison purpose, we also generate another block‐group‐level NO_2_ data by first oversampling TROPOMI NO_2_ to a regular grid of 0.01° × 0.01° using the area‐weighted average approach (hereafter AWO‐Grid), where the weight is determined by the overlapping areas between satellite pixel and each grid cell (Jin et al., 2020). If a block group is greater than the size of a grid cell, we calculate the average over all grid cells within the boundaries of the block group. If a block group is smaller than the size of a grid cell, we assign the NO_2_ with the grid cell nearest to the centroid of the block group (Kerr et al., 2021).

### 2.2 Demographic and Administrative Data

Demographic data are obtained from the National Historical Geographic Information System (Manson et al., 2024). The demographic information is derived from the American Community Survey conducted by the US Census Bureau. We extract 5‐year 2018–2022 estimates on race, ethnicity and income. The block‐group geographic information, including the administrative boundaries, geographic entity codes (GEOIDs) and associated state, county, and census tract names are acquired as Shapefile format from the Topologically Integrated Geographic Encoding and Referencing system of the US Census Bureau (U.S. Census Bureau, 2023). We also acquire the boundaries and area titles information of the US core‐based statistical areas (CBSA, collectively representing metropolitan and micropolitan statistical areas) from the US Census Bureau (U.S. Census Bureau, 2020).

## 3 Results and Discussions

### 3.1 Oversampled NO_2_ Data Set at Neighborhood Scales

By directly oversampling daily TROPOMI NO_2_ observations to over 237,000 block groups over the CONUS using PGO‐BG and AWO‐BG approaches, we develop a neighborhood‐scale TROPOMI‐based NO_2_ data set. The area size of U.S. block groups varies from approximately 100 m^2^ to over 10,000 km^2^, and 43% of them are within 1 km^2^ (Figure S1 in Supporting Information S1). The developed NO_2_ data set is in tabular and GIS compatible formats, searchable by state, county, census tract or neighborhood GEOIDs, which allows for directly queries or selections based on locations of interest. The NO_2_ data set is based on administrative shapes, which could be aggregated to obtain NO_2_ values at higher administrative levels such as census tracts, counties, CBSAs etc. As no ancillary data are needed for producing the data, it can be made available within hours of TROPOMI measurements acquisition. The developed NO_2_ data set is available at multiple temporal resolutions from daily to 5‐year average, which could inform where and when the NO_2_ exposure is highest, offering opportunities for targeted interventions to protect marginalized and vulnerable populations.

**Figure 2:**
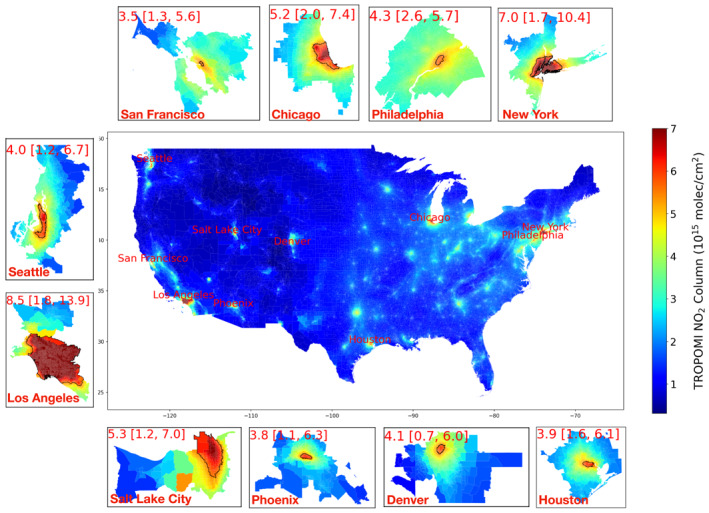
Map of 5‐year average (2019–2023) NO_2_ at block group level over contiguous United States with zoom‐in maps of NO_2_ for 10 CBSAs with the highest level of NO_2_. The block‐group NO_2_ is calculated using PGO‐BG approach. Block groups where NO_2_ exceeds top 10% national level (>5.5 × 10^15^ molecules/cm^2^) are aggregated and outlined in black. The numbers in the zoomed‐in maps represent the mean NO_2_ column density (in unit of 10^15^ molecules/cm^2^), with the values in brackets indicating the minimum and maximum within each core‐based statistical areas.

Figure 2 shows the 5‐year average (2019–2023) block‐group level NO_2_ column density over the CONUS, oversampled with the PGO‐BG approach. The average NO_2_ ranges from 0.3 to 13.9 × 10^15^ molecules/cm^2^ with the maximum values over downtown Los Angeles. Block groups where NO_2_ exceeds top 10% national level (>5.5 × 10^15^ molecules/cm^2^) are labeled in Figure 2, which are located in 10 metropolitan areas, including Los Angeles, New York, Salt Lake City, Chicago, Philadelphia, Seattle, Denver, Phoenix, Houston, and San Francisco. These metropolitan areas show large spatial gradients of NO_2_, where the NO_2_ columns vary by over a factor of 2 (Philadelphia) to 8 (Denver) from the least to the most polluted neighborhoods. Neighborhoods with the highest NO_2_ are mostly located in downtown urban areas with major roads, heavy traffic, or industrial activity hubs (Goldberg et al., 2021). Outside of populated urban areas, we find relatively small NO_2_ variations. This suggests that our proposed oversampling method is suitable for primary pollutants like NO_2_, which captures sharp gradients in populated areas with compact administrative units while maintaining balanced representation in rural regions.

### 3.2 Comparison of Different Spatial Oversampling Methods

**Figure 3:**
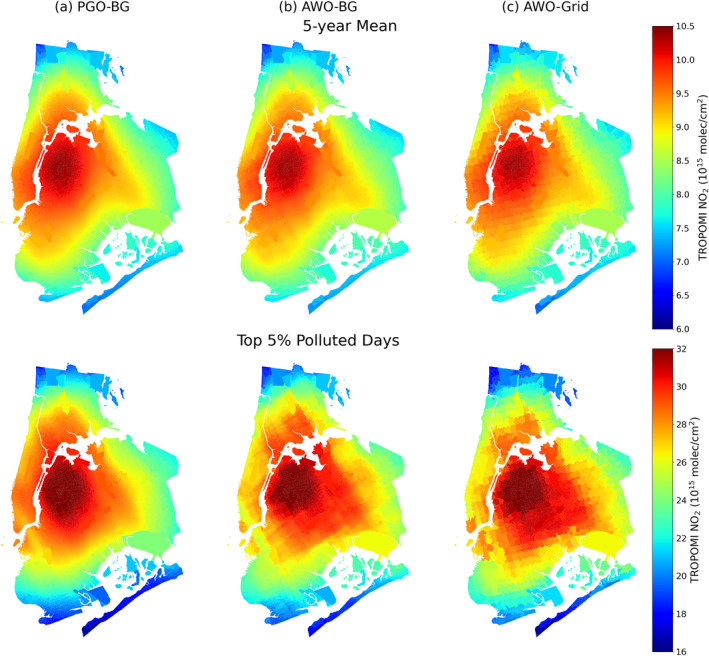
Maps of block‐group level TROPOspheric Monitoring Instrument NO_2_ for New York City based on 5‐year average (upper panel) and top 5% polluted days (bottom panel) using three oversampling methods: (a) Physics‐based Gaussian oversampling to block groups (PGO‐BG); (b) area weighted average oversampling to block groups (AWO‐BG); (c) area weighted average method oversampling to a regular grid of 0.01° × 0.01° (AWO‐Grid).

Next, we assess the extent to which the proposed spatial oversampling methods outperform other commonly used oversampling methods, especially in populated urban areas with sharp gradients of NO_2_. The top panel of Figure 3 compares the block‐group level 5‐year average TROPOMI NO_2_ over New York City generated from three oversampling methods, including the PGO‐BG, AWO‐BG, and AWO‐Grid. The area size of block groups ranges from ∼100 m^2^ to 18 km^2^ in this region with the 99% being smaller than 1 km^2^. Despite that the overall spatial patterns of NO_2_ are similar across the three methods, the two administrative unit‐based methods (PGO‐BG and AWO‐BG) show the smoothest spatial patterns with clear identification the neighborhoods with highest NO_2_ pollution (Figure 3). While a fine resolution of 1 km is used in the gridded approach (AWO‐Grid), we find it causes noisier patterns with a more segmented or “blocky” appearance. The spatial patterns of NO_2_ generated from the gridded data sets are sensitive to the resolution and how the grids are matched to block groups. Two block groups that share the same grid will be assigned with the same NO_2_ values, and a small shift of grids could result in different spatial patterns of NO_2_. Our proposed administrative unit‐based oversampling methods eliminate the need for gridding at a given resolution, meaning that they are resolution‐adaptive, which enhances the resolution and representation of NO_2_ in urbanized areas with compact administrative units. Our proposed administrative unit‐based methods are also more computationally efficient than the gridded method: the total number of target grids at 1‐km resolution is around 14,144,500, which is 60 times more than the total number of target block groups (∼237,000).

Comparing the two administrative unit‐based methods (PGO‐BG and AWO‐BG), PGO‐BG and AWO‐BG show almost identical patterns of NO_2_ with less than 1% difference for the 5‐year average. The AWO‐BG method is more computationally efficient, as the calculation is vector‐based without the need to generate spatial response functions. At shorter time scales, however, PGO‐BG method tends to provide smoother patterns of NO_2_ than AWO‐BG (Sun et al., 2018). The bottom panel of Figure 3 compares the oversampled NO_2_ averaged for the 5% most polluted days (∼40 days), which are selected based on the regional daily mean TROPOMI NO_2_. We find that the PGO‐BG approach better resolves the spatial gradient than the area weighted average approach. As the satellite observations are defined as polygons in the AWO‐BG approach, averaging over a short temporal window results in segmented appearance, and the segmented appearance is even worse when they are oversampled to regular grid (AWO‐Grid, Figure 3c). Another advantage of the PGO‐BG approach is that it reduces the number of missing values. In the AWO‐BG approach, only block groups that overlap with satellite pixel polygons are assigned with values, and missing values of TROPOMI observations caused by cloud or other retrieval issues would result in missing values for all block groups inside this polygon. The PGO‐BG approach considers satellite observations as a Gaussian sensitivity distribution, meaning that groups outside the satellite pixel polygons still carry some weight (e.g., block group 1 and 10 in Figure 1b), and block groups within missing satellite pixel can be assigned with values from surrounding valid satellite pixels, increasing the total number of valid observations included to oversample a given block group.

### 3.3 Widespread NO_2_ Disparities Across CONUS

**Figure 4:**
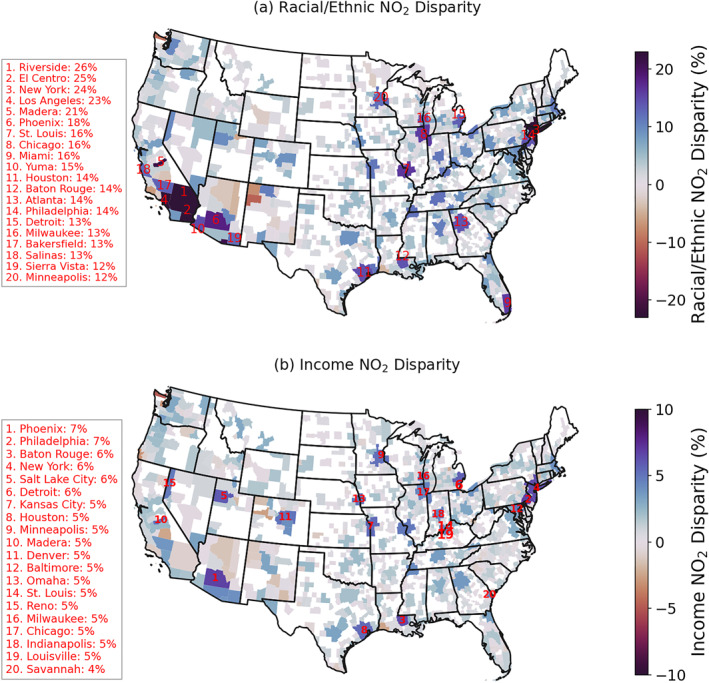
Maps of NO_2_ disparities related to (a) race/ethnicity and (b) income over all CBSAs of contiguous United States derived from block‐group TROPOspheric Monitoring Instrument NO_2_ oversampled with PGO‐BG approach. The racial/ethnic disparity is defined as the relative difference in population‐weighted average NO_2_ between white non‐Hispanic and minority groups (including Hispanic, non‐Hispanic Black, Asian, and native American), and the income‐related disparity is defined as the relative difference in population‐weighted average NO_2_ between the groups with ratio of income to poverty level below 1.24 (hereafter low‐income groups) and the groups with the ratio above 1.5 (hereafter high‐income groups). The left panel shows the top 20 CBSAs with the largest NO_2_ disparity, with numbers on the map indicating their rank. The results using the other two oversampling approaches can be found in Supporting Information S1.

An advantage of the block‐group level NO_2_ data set is its compatibility with demographic and socio‐economic information, suitable for studying the environmental disparity issues related to NO_2_ pollution. Here we analyze the difference in the NO_2_ levels across different racial/ethnic and income groups over 900 CBSAs of the CONUS. For each CBSA, we calculate the population‐weighted average NO_2_ level for different racial/ethnic and income groups using block‐group level TROPOMI NO_2_ and demographic data. We define racial/ethnic NO_2_ disparity as the relative difference in population‐weighted average NO_2_ between white non‐Hispanic and minority groups (including Hispanic, Black, Asian, and Native American). Figure 4a shows the TROPOMI‐derived racial/ethnic NO_2_ disparity at CBSA level, calculated using PGO‐BG oversampling approach. We find more than 80% CBSAs show that racial/ethnic minority groups experiencing higher level of NO_2_. The top 20 metropolitan areas with largest NO_2_ disparity are labeled in Figure 4a. Metropolitan areas such as Los Angeles, New York City, Chicago are also among the regions with highest levels of NO_2_ (Figure 2) and population density. We find the largest NO_2_ racial/ethnic disparity over Southern California, including Los Angeles, Riverside, El Centro and Madera areas, where minority groups experience more than 20% higher NO_2_ than white non‐Hispanic groups. Figure S2 in Supporting Information S1 shows the racial composition at different levels of NO_2_ over Los Angeles metropolitan area. Regions with the highest NO_2_ pollution, mostly located in downtown Los Angeles, are dominated by Hispanic communities. Other statistical areas with large racial/ethnic NO_2_ disparities include New York City (24%), Phoenix (18%), Saint Louis (16%), Chicago (16%), Miami (16%), and Yuma (15%). Our results are consistent with prior studies that minority population is exposed to higher level of NO_2_ pollution than the non‐Hispanic white population (Bluhm et al., 2022; Clark et al., 2014, 2017; Demetillo et al., 2021; Hrycyna et al., 2022; Kerr et al., 2021; Y. Wang et al., 2023). Most statistical areas with high NO_2_ levels (>3 × 10^15^ molecules/cm^2^) show clear NO_2_ disparities (>5%). However, a few exceptions exist, including Trenton (NJ), New Haven (CT), Boulder and Greeley (CO), and Portland (OR), where the relative NO_2_ disparities are below 5%. In Trenton and New Haven, NO_2_ disparities are still present, but elevated NO_2_ levels are largely influenced by regional transport of NO_x_ emissions from and nearby big cities (e.g., New York and Philadelphia) and interstate highways. The low disparities in Portland, Boulder, and Greeley reflect smaller overall minority populations and less pronounced patterns of residential segregation.

**Figure 5:**
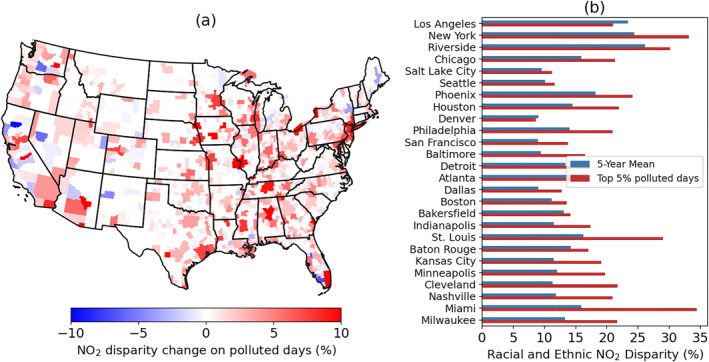
(a) Difference in the racial/ethnic NO_2_ disparities between the top 5% polluted days and the 5‐year mean. (b) Comparison of the racial/ethnic NO_2_ disparities on the top 5% polluted days versus the 5‐year mean NO_2_ in selected CBSAs. NO_2_ data are oversampled with the PGO‐BG approach. The top 5% days are selected based on the daily mean TROPOMI NO_2_ at each core‐based statistical areas. The CBSAs are ranked by the 5‐year mean NO_2_ level with Los Angeles being the highest. The results using the other two oversampling approaches can be found in Supporting Information S1.

Figure 4b shows the income‐related NO_2_ disparity, defined as the percentage difference in population‐weighted average NO_2_ between the groups with ratio of income to poverty level below 1.24 (hereafter low‐income groups) and the groups with the ratio above 1.5 (hereafter high‐income groups) (Demetillo et al., 2021). We find similar widespread NO_2_ disparities across CONUS: 75% CBSAs show that low‐income communities are exposed to higher level of NO_2_ than high‐income communities, but the magnitudes of income‐related disparities are overall smaller than that of race/ethnicity. The top 20 CBSAs with largest income‐related NO_2_ disparities are labeled in Figure 5b. We find the largest income‐related NO_2_ disparities over Phoenix (6.8%), Philadelphia (6.7%), Baton Rouge (6.4%), New York City (6.4%) and Salt Lake City (5.9%) metropolitan areas. The CBSAs with large income‐related NO_2_ disparities also show large racial/ethnic NO_2_ disparities, but not all CBSAs with large race/ethnicity related NO_2_ disparities also show income related NO_2_ disparities. For example, while Southern California shows the highest NO_2_ disparity between white non‐Hispanic and minority racial groups, we find little difference (2.3%) in NO_2_ exposure between high‐ and low‐income groups. Similarly, we find small income‐related NO_2_ disparities in Miami and Atlanta, despite large racial/ethnic NO_2_ disparities. Such findings are consistent with previous studies that suggest racial/ethnic disparities are larger than the income‐related disparities, suggesting that racial/ethnic disparities are not merely a reflection of income difference among racial/ethnic groups (Kerr et al., 2021; Liu et al., 2021).

Next, we compare the NO_2_ disparity estimates across the three oversampling approaches. The results from the physics‐based Gaussian method (PGO‐BG) closely align with those from the two area‐weighted methods (AWO‐BG and AWO‐Grid), showing consistent estimates of racial/ethnic and income‐related NO_2_ disparities with differences generally within 2% (Figures S3 and S4 in Supporting Information S1). As discussed in Section 3.2, the long‐term average NO_2_ patterns are almost identical between the PGO‐BG and AWO‐BG. Also, the NO_2_ disparities identified here are largely structured at the regional scale rather than the fine‐grained block‐group level. As a result, the long‐term NO_2_ disparities are insensitive to the choice of spatial oversampling methods.

### 3.4 Increased NO_2_ Disparities During Polluted Days

NO_2_ disparities reported above mainly focus on the long‐term average of NO_2_, most relevant to the chronic health effects of NO_2_. Short‐term exposure to high levels of NO_2_ has shown to be associated with increasing total, cardiovascular and respiratory mortality (Meng et al., 2021). We show that PGO‐BG oversampling is advantageous for shorter temporal windows (Section 3.2), useful for studying the short‐term variability of NO_2_ disparities. Here, we focus on evaluating NO_2_ disparity at the upper end of NO_2_ distribution, when NO_2_ pollution has the most significant acute health effects (R. Chen et al., 2012; He et al., 2020; Meng et al., 2021). As we focus on relative disparity rather than the absolute difference, the level of NO_2_ disparity should be independent of the absolute NO_2_ level (Harper et al., 2013). Figure 5a shows the difference in racial/ethnic NO_2_ disparities across all CBSAs, calculated as the NO_2_ disparity on the top 5% polluted days minus the 5‐year average. Overall, NO_2_ disparities tend to be higher on the most polluted days compared to the long‐term average. Out of 747 statistical areas where positive NO_2_ disparities are found on 5‐year basis, 576 (77%) show an increase in NO_2_ disparities on polluted days, 75 of them show an increase above 5% (Figure 5a). The increase of NO_2_ disparities is apparent over the most polluted metropolitan areas, such as New York and Chicago (Figure 5b). We find the largest increase in Miami, where the racial/ethnic NO_2_ disparity increases by a factor of two from 16% to 34% during the polluted days (Figure 5a). St. Louis shows the second largest increase of racial/ethnic NO_2_ disparity from 16% to 29% (Figure 5a). The income‐related NO_2_ disparities also increase on polluted days (Figure S5 in Supporting Information S1). As the health effects of NO_2_ increases with NO_2_ levels (He et al., 2020; Meng et al., 2021), the overall increase of NO_2_ disparities implies greater acute health effects for overburdened communities.

**Figure 6:**
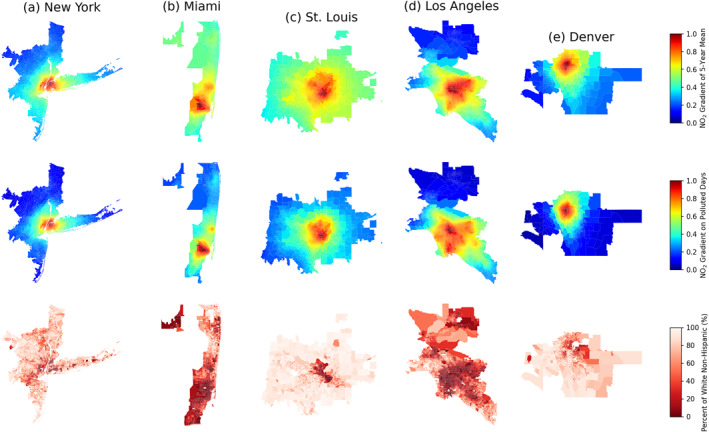
Normalized spatial gradient of NO_2_ in selected CBSAs: (a) New York; (b) Miami; (c) St. Louis; (d) Los Angeles and (e) Denver based on 5‐year average (top panel) versus top 5% polluted days (middle panel), calculated as the block‐group NO_2_ divided by the maximum NO_2_, using the PGO‐BG approach to oversample NO_2_. The bottom panel shows the percentage of white non‐Hispanic population. The extent of each map is defined based on the full extent of the corresponding core‐based statistical areas, which include the city labeled and the surrounding areas.

Days with the highest NO_2_ pollution often occur in winter, when NO_2_ has a long chemical lifetime, and the thermal inversions cause it to be trapped near the emission sources that are often located in overburdened communities (Wallace & Kanaroglou, 2009; S.‐Y. S. Wang et al., 2015). Figure 6 shows the normalized spatial gradient of NO_2_ in selected urban areas during the top 5% polluted days versus 5‐year average, where the spatial gradient of NO_2_ is defined as the block‐group NO_2_ divided by the maximum NO_2_ in given area. On polluted days, we observe sharper NO_2_ gradients, which cause larger difference of NO_2_ between white non‐Hispanic and minority dominated communities. For example, in greater New York metropolitan area, the NO_2_ level in surrounding suburban area is about 10% of the level in downtown Manhattan but is around 40% on 5‐year average (Figure 6a). Dressel et al. (2022) also find that daily NO_2_ inequalities over New York are negatively correlated with wind speed. We find the largest increase of NO_2_ disparity on polluted days over Miami and St. Louis, not only because they show sharper NO_2_ gradient during the polluted days, but also clear regional patterns of residential segregation by race/ethnicity that make these two cities sensitive to the changes of NO_2_ gradient (Figures 6b and 6c). However, not all cities show increased NO_2_ disparities on the most polluted day. Two exceptions are Los Angeles and Denver, where NO_2_ disparities are overall smaller on top 5% polluted days (Figures 6d and 6e). As shown in Figure 6d, NO_2_ in Los Angeles is more dispersed during the top 5% polluted days, and we observe an outflow of NO_2_ from downtown LA to the West Side and South Bay regions, where the population is dominated by white non‐Hispanic population. In Denver (Figure 6e), the center of peak NO_2_ was observed along the North Valley Highway over North Denver based on 5‐year mean, where the population is dominated by minority groups, but the center has moved southward toward the central Denver during the top 5% polluted days.

Next, we compare the changes of NO_2_ disparities on polluted days across the three spatial oversampling methods. We find that the overall increase in NO_2_ disparities is not captured by the two area‐weighted oversampling methods (Figures S6–S9 in Supporting Information S1). Only 21 statistical areas show an increase greater than 5% in racial/ethnic NO_2_ disparities on polluted days when using the AWO‐BG approach, and 19 areas show such an increase with the AWO‐Grid approach. In areas like New York and Chicago, decreased NO_2_ disparities are found during the top 5% polluted days with the AWO‐BG (Figures S6 and S8 in Supporting Information S1) and AWO‐Grid approaches (Figures S7 and S9 in Supporting Information S1). At short temporal windows, the spatial patterns of NO_2_ are sensitive to the choice of oversampling approach (Figure 3), and the selection of the polluted days is affected by missing observations in which PGO‐BG has fewer missing values (Section 3.2), which together leading to differences in the NO_2_ disparities on polluted days between physics‐based and area weighted oversampling approaches.

## 4 Conclusions

Satellite observations provide continuous and full‐coverage observations of air pollutants such as NO_2_, which have been widely used to inform health impacts and air pollution disparity. Linking satellite‐retrieved air pollution data with socioeconomic or health data involves matching the swath satellite observations with administrative units. Here we use both physics‐based Gaussian (PGO‐BG) and area weighted average (AWO‐BG) approaches to spatially oversample TROPOMI NO_2_ observations directly to administrative shapes at block‐group level. Comparing with the traditionally used gridded approach (AWO‐Grid), we show that directly oversampling satellite observations to administrative shapes is a more accurate and computationally efficient approach for integrating satellite observations with socioeconomic and health data. For users interested in long‐term average NO_2_, AWO‐BG approach is recommended because the vector‐based calculation is more computationally efficient, and it results in almost identical spatial patterns as the PGO‐BG approach. However, for users interested in studying the short‐term variability of NO_2_, we show that the PGO‐BG approach is advantageous, which better captures the spatial variability of NO_2_ and results in less missing values than AWO‐BG.

Using the newly developed NO_2_ data set, we analyze the racial/ethnic and income‐related disparities of NO_2_ over 900 statistical areas of CONUS. We find that more than 80% CBSAs show racial/ethnic minority groups experiencing higher level of NO_2_, and populated metropolitan areas such as Los Angeles, New York, and Chicago are among the areas with the largest NO_2_ level as well as NO_2_ disparities. We also find widespread income‐related NO_2_ disparities (>75% of CBSAs), but the magnitude is overall smaller, and not all areas with racial/ethnic disparity show income‐related disparities. Contrasting the NO_2_ disparities on the top 5% polluted days with the normal conditions, we find that the NO_2_ disparities generally increase on the most polluted days due to the accumulation of emissions under stagnation events. The increased disparities during polluted days imply larger acute health effects for overburdened communities.

Here we aim to provide descriptive, observation‐based evidence of air pollution disparities. Causal attribution of the disparities to policy and socioeconomic factors is beyond scope of this study but has been extensively discussed in literature (Bluhm et al., 2022; Currie et al., 2023; Hrycyna et al., 2022). While the physics‐based spatial oversampling methods allow us to resolve the spatial heterogeneity beyond the native resolution of TROPOMI, they do little to enhance the true resolving power of the satellite observations after reaching a certain resolution (Sun et al., 2018). Improved characterization of NO_2_ heterogeneity will benefit from future improvements of satellite instrument resolution, the expansion of monitoring network, as well as inclusion of ancillary information such as traffic and road density. Also, the NO_2_ disparities are quantified based on tropospheric NO_2_ columns, different from the surface mixing ratio regulated by US EPA. While a strong correlation is found between TROPOMI NO_2_ column and surface NO_2_ (Goldberg et al., 2021), their relationship is influenced by the boundary layer height (Jin et al., 2017) and contributions from free tropospheric NO_2_ (Shah et al., 2022). Also, the overpass time of TROPOMI is around 1:30 p.m. local time, when NO_2_ concentration and lifetime is typically lowest throughout the day, and thus the disparities of NO_2_ may be even higher at other times of the day. NASA's Tropospheric Emissions: Monitoring of Pollution (TEMPO), a geostationary satellite mission launched in 2023, provides retrievals of NO_2_ with hourly frequency over North America during daylight hours, which will allow for detailed characterizations of the diurnal variations of NO_2_ disparities (Chance et al., 2013; Gonzalez Abad et al., 2024).

## Conflict of Interest

The authors declare no conflicts of interest relevant to this study.

## Supporting information

Supporting Information S1

## Data Availability

Level‐2 TROPOMI NO_2_ data are obtained from Tropospheric Emission Monitoring Internet Service (TEMIS) hosted by Royal Netherlands Meteorological Institute (Copernicus Sentinel‐5P, 2021). Demographic data are obtained from the National Historical Geographic Information System (Manson et al., 2024). The block‐group geographic information is accessible from Topologically Integrated Geographic Encoding and Referencing system of US Census Bureau (U.S. Census Bureau, 2023). Boundaries and area titles information of US CBSA is accessible from US Census Bureau (U.S. Census Bureau, 2020). The processed block‐group level NO_2_ data along with demographic information are available in Jin (2025).
