# Supplementary material for: Physics‐Based Spatial Oversampling of TROPOMI NO2 Observations to US Neighborhoods Reveals the Disparities of Air Pollution
Source: Geohealth. 2025 Aug 16;9(8):e2025GH001423. doi: 10.1029/2025GH001423 (PMC12357153; doi:10.1029/2025GH001423)
Supplement: Supplementary file 1 — Supporting Information S1 [file GH2-9-e2025GH001423-s001.pdf]

GeoHealth

Supporting Information for

Physics-based spatial oversampling of TROPOMI NO_2 observations to US neighborhoods reveals the disparities of air pollution

Xiaomeng Jin[1*], Zaina Merchant[1], Kang Sun[2]

1. Department of Environmental Sciences, Rutgers, The State University of New Jersey, New Brunswick, NJ, USA
2. Department of Civil, Structural and Environmental Engineering, University at Buffalo, Buffalo, NY, USA

Corresponding Author's Email Address: xiaomeng.jin@rutgers.edu

Contents of this file

Figures S1 to S9

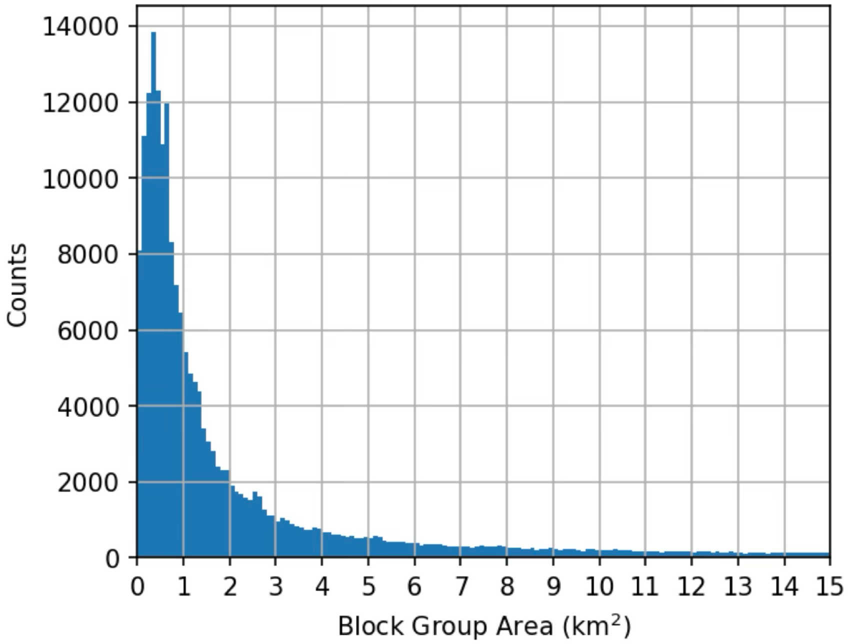

Figure S1 Distribution of the area of block groups over CONUS.

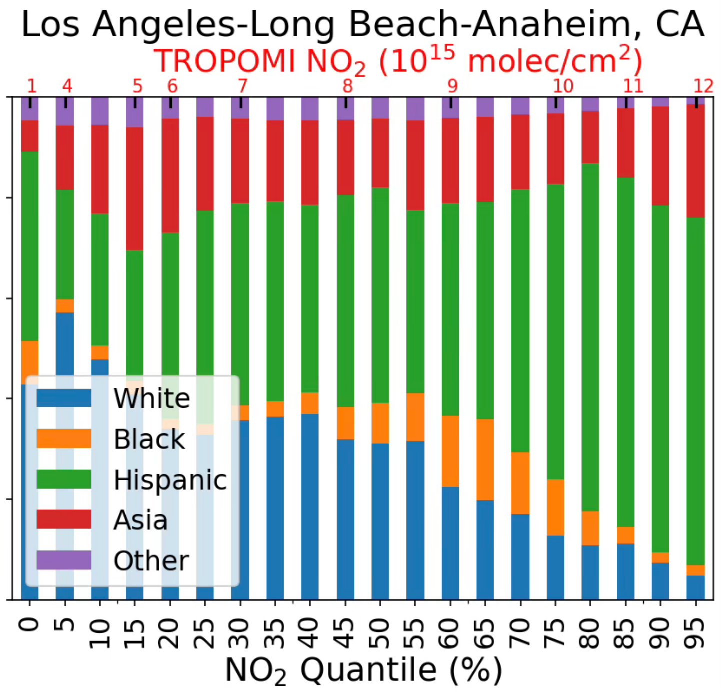

Figure S2 Racial and ethnic composition as a function of NO_2 level over Los Angeles area. The number on the top shows the corresponding TROPOMI NO_2 level.

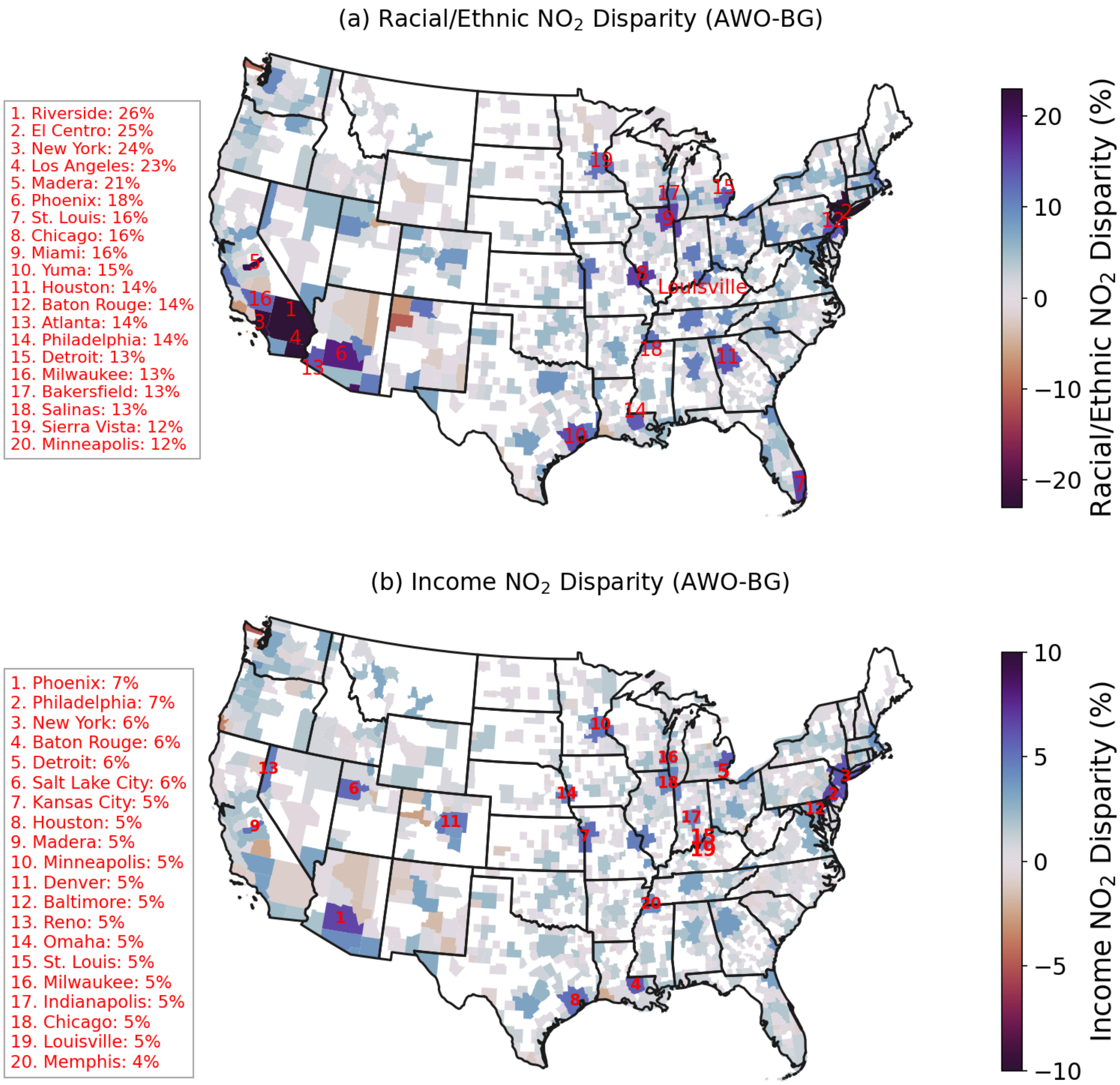

Figure S3 Same as Figure 4 but is based on TROPOMI NO_2 data oversampled using the block-group based area weighted average approach.

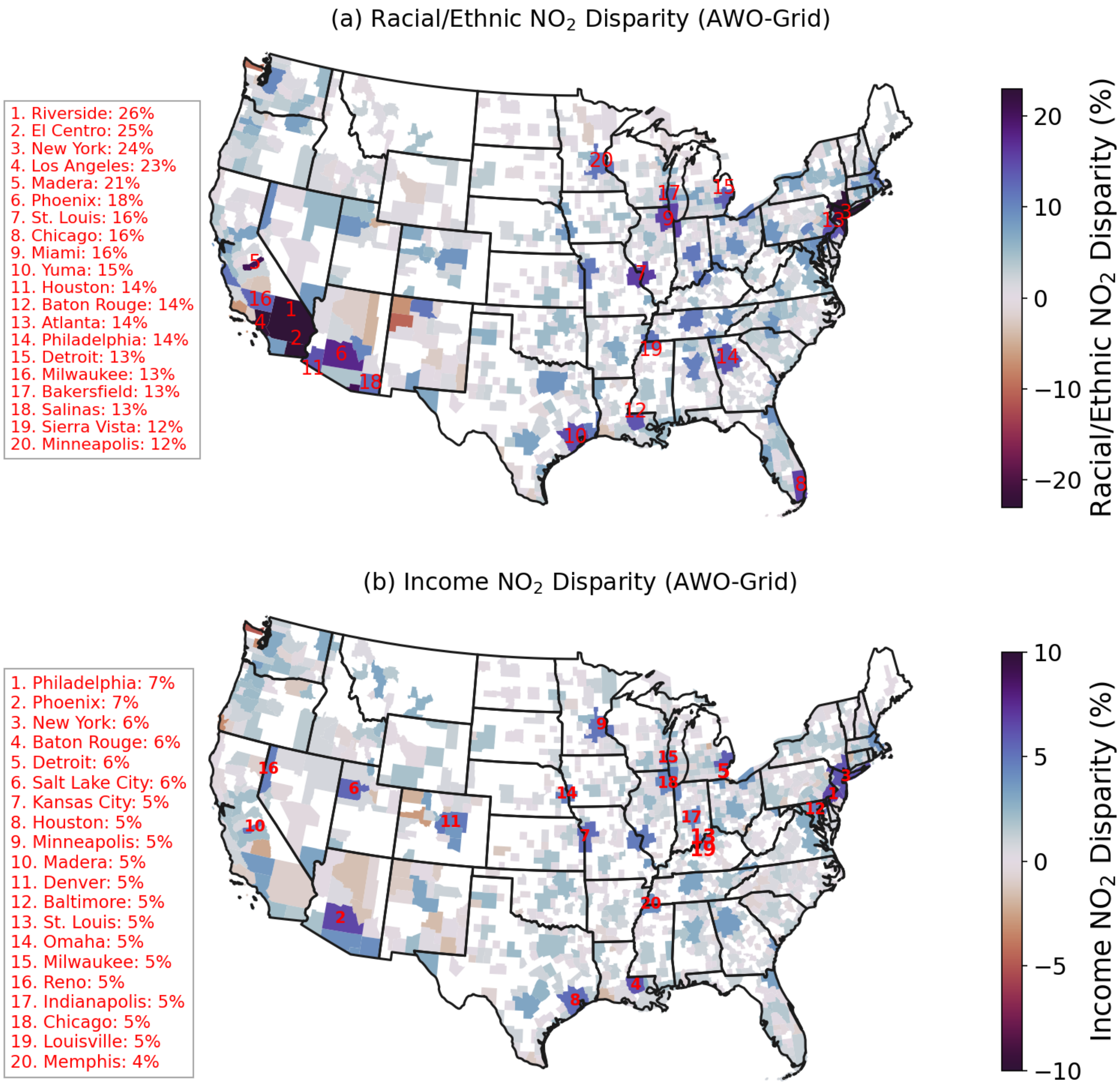

Figure S4 Same as Figure 4 but is based on TROPOMI NO_2 data oversampled using the gridded approach.

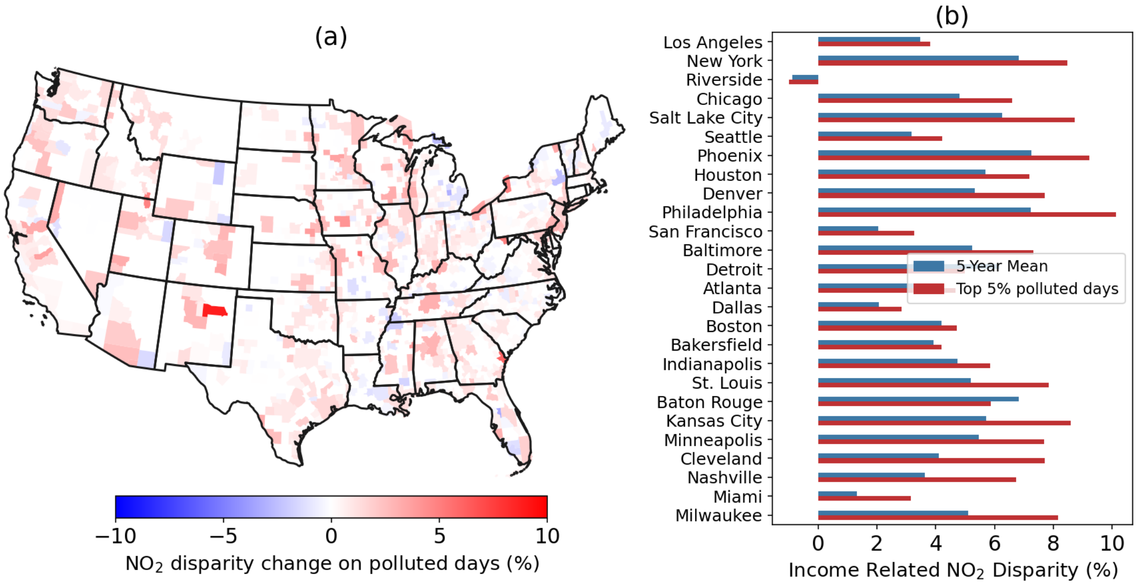

Figure S5 Same as Figure 5 but for income-related NO_2 disparity.

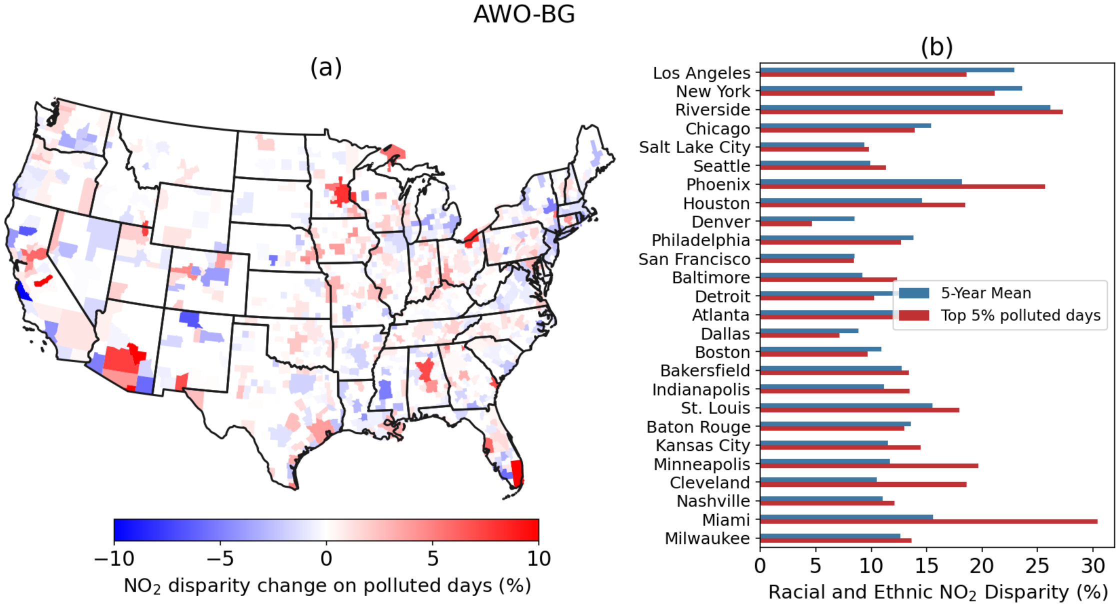

Figure S6 Same as Figure 5 but is based on TROPOMI NO_2 data oversampled using the block-group based area weighted average approach.

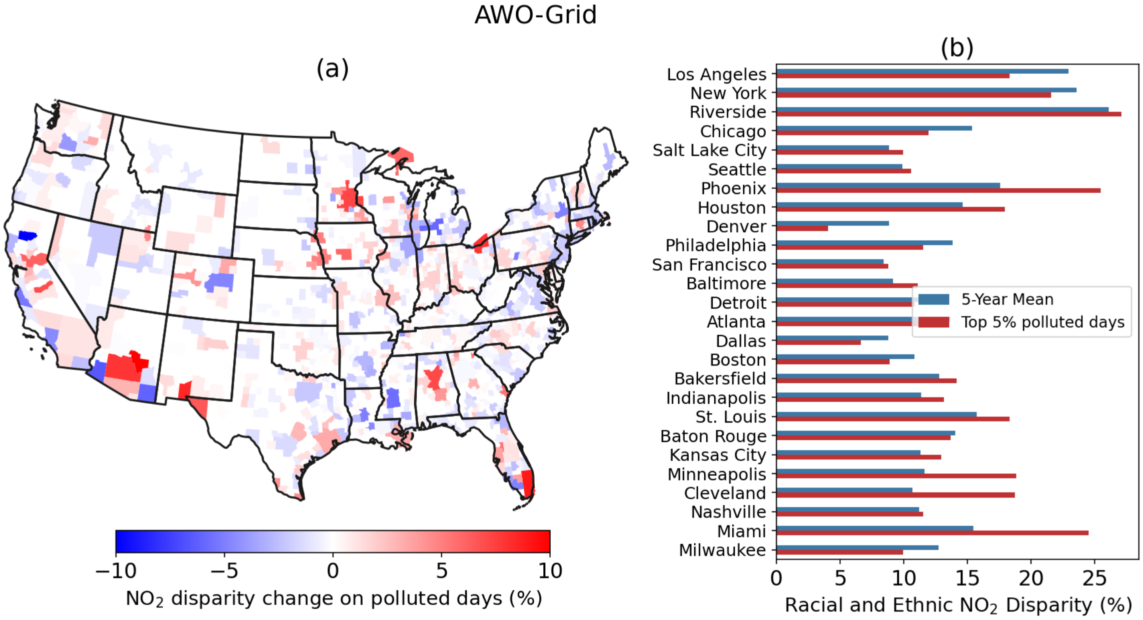

Figure S7 Same as Figure 5 but is based on TROPOMI NO_2 data oversampled using the gridded approach.

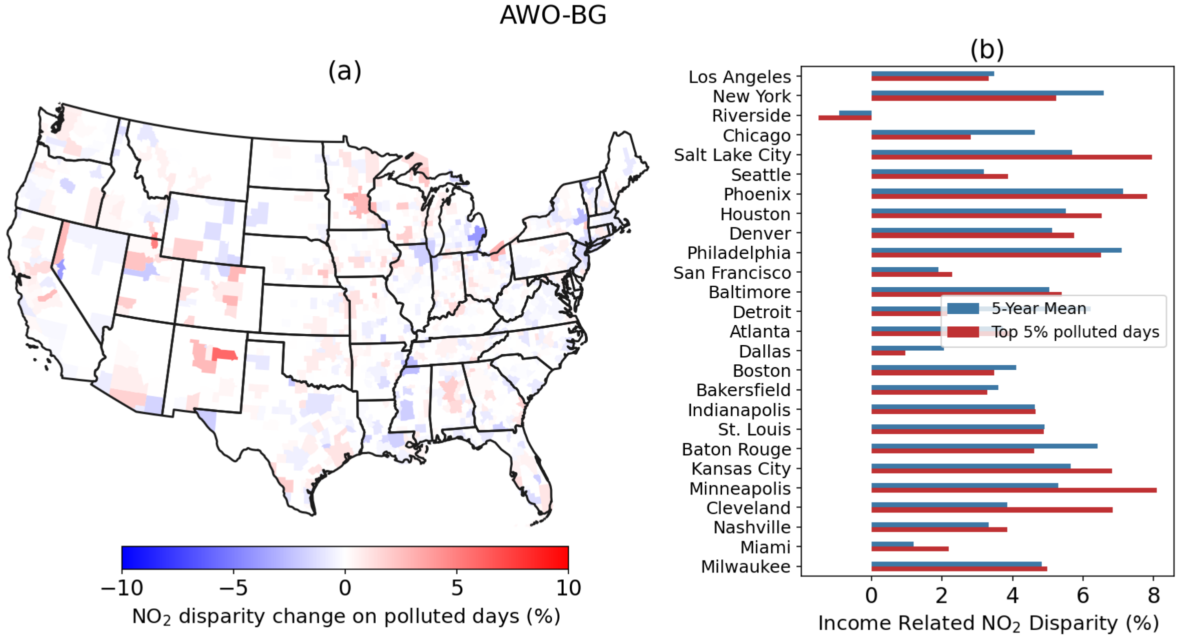

Figure S8 Same as Figure S5 but is based on TROPOMI NO_2 data oversampled using the block-group based area weighted average approach.

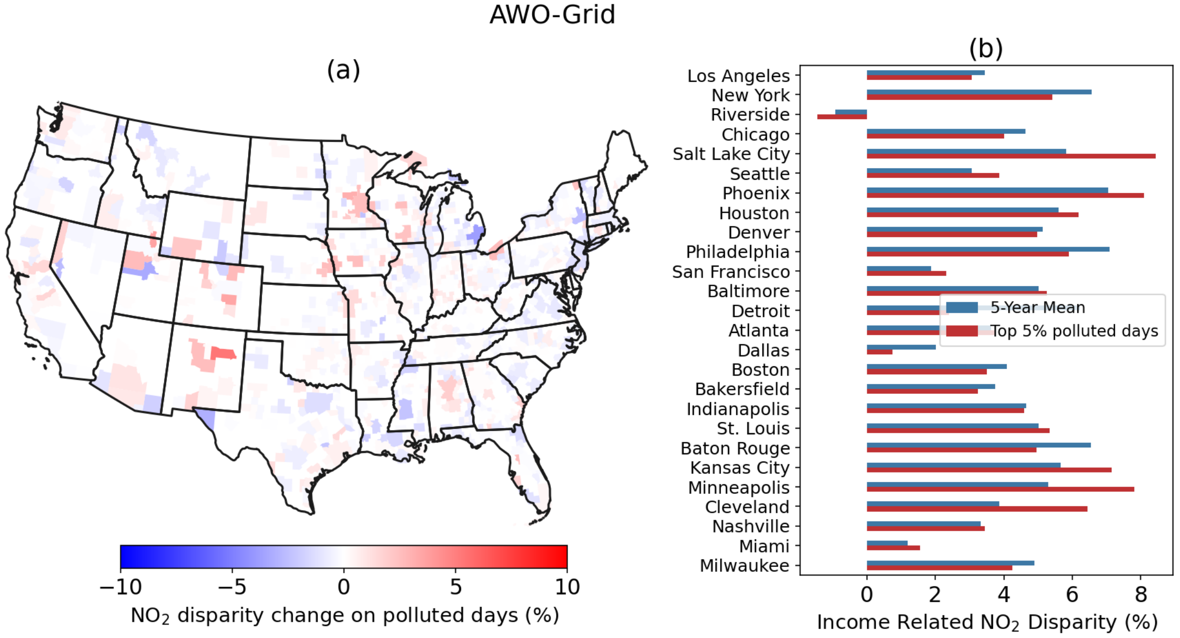

Figure S9 Same as Figure S5 but is based on TROPOMI NO_2 data oversampled using the gridded approach.
